# Algorithm‐Defined Muscle Dysmorphia Estimates Across Weighting and Case‐Definition Strategies in a Gender‐Balanced Adult Online Sample

**DOI:** 10.1002/eat.70170

**Published:** 2026-07-09

**Authors:** Christopher Zaiser, Nora M. Laskowski, Georg Halbeisen, Marietta Lieb, Georgios Paslakis

**Affiliations:** ^1^ Clinic for Psychosomatic Medicine and Psychotherapy LWL‐University Clinic, Ruhr‐University Bochum Bochum Germany; ^2^ Faculty of Business Administration and Economics OWL University of Applied Sciences and Arts Lemgo Germany

**Keywords:** body image, case definition, eating disorders, epidemiology, gender differences, muscle dysmorphia, online sample, prevalence, psychometrics, weighting

## Abstract

**Objective:**

Epidemiological evidence on muscle dysmorphia (MD) remains limited, and self‐report algorithm‐defined estimates may depend on sampling and case definitions. We examined how algorithm‐defined MD estimates and exploratory correlates varied across weighting and case‐definition scenarios in a gender‐balanced German online sample.

**Method:**

In this cross‐sectional web‐based study, 1468 adults from Germany completed self‐report measures: 739 (50.3%) men, 706 (48.1%) women, 21 (1.4%) nonbinary/diverse participants, and 2 (0.1%) who preferred not to disclose their gender. Algorithm‐defined MD was estimated using a self‐report algorithm derived from prior epidemiological work. Estimates were compared across four scenarios combining unweighted versus age‐ and gender‐weighted analyses with global versus gender‐specific criterion A cutoffs. Logistic regression models examined correlates across weighted and unweighted analytic specifications.

**Results:**

Algorithm‐defined estimates varied substantially across operationalizations. The unweighted global algorithm yielded an estimate of 6.2% overall and 11.0% in men. The weighted gender‐specific scenario yielded 2.6% overall, 3.5% in men, and 1.7% in women. Across regression specifications, lower BMI and higher identity disturbance were the most consistent correlates of algorithm‐defined MD. Female gender showed lower odds in pooled models, particularly under the global cutoff, but this finding should be interpreted cautiously.

**Discussion:**

Self‐report algorithm‐defined MD may affect a meaningful minority of adults, but estimates depend strongly on weighting strategy and case definition. These findings highlight the need for transparent reporting of algorithmic operationalizations.

## Summary

- This study examined algorithm‐defined MD estimates in a gender‐balanced adult online sample from Germany.
- Estimates varied depending on whether analyses were weighted and whether global or gender‐specific cutoffs were used.
- The weighted gender‐specific scenario yielded an estimate of 2.6% overall, 3.5% in men, and 1.7% in women, illustrating the sensitivity of estimates to analytic choices.
- Lower BMI and greater identity disturbance were the most consistent correlates of algorithm‐defined muscle dysmorphia.

## 1 Introduction

Research on disordered eating and body image psychopathology has historically focused primarily on thinness‐oriented concerns, particularly among women (Paslakis 2026). In recent decades, however, increasing attention has been directed toward muscle dysmorphia (MD), a clinically relevant condition characterized by a persistent belief of being insufficiently muscular or lean despite average or high muscularity (Halbeisen et al. 2022; Halbeisen et al. 2024; Mitchison et al. 2022). Individuals with MD often engage in rigid dietary practices, excessive resistance training, and compulsive appearance‐related behaviors to attain an idealized physique; some also use appearance‐ and performance‐enhancing drugs (APEDs) (Ganson, Testa, et al. 2025; Martenstyn et al. 2022, 2023; Pope et al. 1997). Although MD is included as a specifier of body dysmorphic disorder in DSM‐5 (American Psychiatric Association 2013) and is described within the ICD‐11 clinical descriptions for body dysmorphic disorder (World Health Organization 2022), its phenomenology overlaps substantially with eating disorder (ED) psychopathology, and some authors have advocated for a more differentiated conceptualization (Ganson, Testa, et al. 2025; Murray et al. 2010).

MD is clinically relevant because it is associated with distress, functional impairment, and potentially harmful behavioral patterns (Çınaroğlu and Yılmazer 2025; Laskowski et al. 2025; Murray et al. 2010; Pope et al. 2017; Zaiser et al. 2024). Elevated depressive, anxiety, and obsessive‐compulsive symptoms further underscore MD's burden (Chandler et al. 2009; Mitchell et al. 2017). Because MD often emerges during adolescence and early adulthood but is not confined to these developmental periods, population‐level estimates are important for screening, prevention, and intervention planning (Mitchison et al. 2022; Nieuwoudt et al. 2015; Pope et al. 1997; Strobel et al. 2020).

Epidemiological evidence on MD remains limited, and prevalence estimates vary considerably across studies depending on sample characteristics, assessment strategy, and case definition. Earlier studies in high‐risk groups, particularly bodybuilders, weightlifters, and fitness‐oriented samples, reported prevalence estimates ranging from approximately 7% to more than 17% (Lechner et al. 2019; Nieuwoudt et al. 2015). In Germany, Zeeck et al. (2018) likewise reported comparatively high rates of participants classified as being at risk for MD in a fitness‐oriented community sample, with 25% of men and 16% of women exceeding the questionnaire‐based cutoff used in that study. These estimates likely reflect both the high‐risk character of the sample and the use of questionnaire‐based cutoffs. By contrast, more recent population‐based studies have used algorithm‐based approaches, referring to rule‐based self‐report classifications that combine multiple components corresponding to proposed MD criteria, including muscularity‐related preoccupation, distress or impairment, and differentiation from probable EDs, rather than relying on a single questionnaire total‐score cutoff. Such classifications should be understood as epidemiological approximations rather than clinical diagnoses. Studies using this approach have yielded lower and more convergent estimates, including 2.2% among boys and 1.4% among girls in Australia (Mitchison et al. 2022), and 2.8% in a North American sample of male adolescents and young adults (Ganson, Mitchison, et al. 2025).

An important concern is how algorithm‐defined MD, absent a diagnostic interview, should be operationalized in mixed‐gender samples. Recent algorithm‐based epidemiological applications have been conducted primarily in male or male‐dominated samples (Ganson, Mitchison, et al. 2025; Mitchison et al. 2022), yet data from mixed‐gender studies suggest that women should not be excluded from MD research, even if prevalence and symptom presentation may differ by gender (Mitchison et al. 2022). Recent work suggests that muscularity concerns may be clinically relevant in women (Jürgensen et al. 2025). This raises a methodological question that has received little attention: whether criterion A of the previously applied MD algorithm, operationalized here as muscularity‐related preoccupation via the Drive for Size subscale of the Muscle Dysmorphic Disorder Inventory (MDDI), should be defined using a single global cutoff or gender‐specific cutoffs in mixed adult samples. Existing psychometric work does not resolve this question directly, but it suggests that MDDI subscale distributions, particularly Drive for Size, may vary across gender groups and that norms or thresholds should not automatically be assumed to generalize across samples (Compte et al. 2021; Nagata et al. 2021; Zeeck et al. 2018). Notably, Mitchison et al. (2022) operationalized criterion A in a mixed‐gender adolescent sample using an item developed by the researchers assessing time occupied by thoughts of being too small or not muscular enough, whereas Ganson, Mitchison, et al. (2025) applied an algorithm based on a cutoff in a sample of boys and men. To our knowledge, the implications of applying a cutoff‐based criterion A definition in a mixed‐gender adult sample have not yet been examined directly.

A further methodological issue concerns sample composition. Prevalence estimates derived from online community samples may be biased when the unweighted sample differs from the underlying population on characteristics related to the outcome of interest, particularly under conditions of self‐selection and nonprobability recruitment (Bethlehem 2010; Cornesse et al. 2020; Elliott and Valliant 2017). More broadly, post‐stratification weighting and related adjustment approaches are commonly recommended when nonprobability samples are used to approximate target‐population distributions on variables plausibly related to the outcome (Dever et al. 2008; Yap et al. 2022). In addition, prior research suggests that algorithm‐defined MD may be associated with sociocultural and psychological factors such as internalization of appearance ideals, body dissatisfaction, identity‐related difficulties, attachment insecurity, and APEDs use (Bégin et al. 2019; Convertino et al. 2022; Fabris et al. 2018; Rica and Sepúlveda 2024; Laskowski et al. 2025; Zaiser et al. 2024). Network‐analytic work in men further suggests that muscularity concerns are embedded in a broader symptom structure of disordered eating and body‐shaping behaviors rather than representing an isolated phenomenon (Eschrich et al. 2025). However, these correlates have rarely been examined alongside methodological decisions about case definition in community samples.

Importantly, the present study does not resolve the diagnostic validity of self‐report algorithms for MD; clinical assessment remains necessary for confirmed MD. Rather, it examines how prevalence estimates from currently used algorithmic approaches vary with weighting strategy and criterion A operationalization, making the consequences of these operational and sampling decisions more transparent in a mixed‐gender adult sample.

This primarily descriptive, hypothesis‐generating study addressed two aims. First, we examined algorithm‐defined MD estimates in a gender‐balanced German adult online sample across four scenarios, contrasting global versus gender‐specific cutoffs and unweighted versus age‐ and gender‐weighted analyses. Second, we explored sociodemographic, behavioral, and psychosocial correlates across these specifications to assess whether the association pattern was stable.

## 2 Method

### 2.1 Study Design and Participants

This cross‐sectional web‐based survey was conducted in Germany between July 2025 and January 2026 and was preregistered on AsPredicted (#235,974). The study was approved by the Ethics Committee of the Medical Faculty of Ruhr‐University Bochum (16 April 2025; reference: EK‐Antrag MD Prolific v1.0). Data collection took place via Prolific; participants received £7, approximately €8.20, for completion.

Eligibility criteria were age ≥ 18 years, sufficient German language proficiency, legal capacity to consent, and provision of electronic informed consent. Participation was voluntary and anonymous. The survey was hosted on a secure university server and implemented in jsPsych (De Leeuw et al. 2023). Data were stored pseudonymously and transmitted only after survey completion.

After data cleaning, the full cleaned sample comprised 1468 adults. This sample was used for unweighted descriptive and prevalence analyses. Analyses involving post‐stratification weights were restricted to participants identifying as male or female and providing complete information on the weighting variables because weighting was based on age‐by‐gender population margins and the number of participants in other gender categories was too small for stable weighted estimation.

### 2.2 Measures

All questionnaires were administered in German. Participants reported *age*, *gender identity*, *sexual orientation*, *migration background*, *education*, *relationship status*, *living situation*, *height*, and *weight*; *BMI* was calculated from self‐reported height and weight. Sex assigned at birth and race/ethnicity were not assessed; migration background and education were reported as contextually relevant demographic indicators. Migration background indicated whether the participant or at least one parent had not been born with German citizenship.

*MD symptoms* were assessed with the Muscle Dysmorphic Disorder Inventory (MDDI; Zeeck et al. 2018), with its Drive for Size subscale used to operationalize criterion A of algorithm‐defined MD. *Muscularity‐oriented attitudes and behaviors* were assessed with the Drive for Muscularity Scale (DMS; McCreary and Sasse 2000; Waldorf et al. 2014). *Eating disorder symptoms* were assessed with the Eating Disorder Examination‐Questionnaire (EDE‐Q; Hilbert et al. 2012), and *general psychological distress* with the Kessler Psychological Distress Scale (K10; Kessler et al. 2002). *Additional psychosocial correlates* included obsessive‐compulsive symptoms (DOCS‐SF; Kuehne et al. 2021), self‐concept and identity disturbance (SCIM; Kaufman et al. 2015), attachment insecurity (VASQ; Bifulco et al. 2003), gender‐role orientation (TMF; Kachel et al. 2016), perceived everyday discrimination (EDS; Williams et al. 1997), and supplement use. Present‐sample internal consistencies and descriptive statistics are reported in Table S1.

### 2.3 Data Processing and Analytic Samples

Data were exported from the survey platform and processed in R (version 4.4.1; R Core Team 2024). Because only completed surveys were stored on the server, no partial records were available. Data processing included recoding questionnaire responses, computing scale scores, deriving BMI, and constructing algorithm‐based variables for algorithm‐defined MD and probable EDs.

### 2.4 Statistical Analyses

All analyses were conducted in R. The statistical workflow comprised four components: operationalization of algorithm‐defined MD and probable ED diagnoses, estimation of unweighted and weighted prevalence, weighted descriptive comparisons between participants with and without algorithm‐defined MD, and weighted logistic regression models. All inferential tests were two‐sided with *α* = 0.05. Odds ratios (ORs) with 95% confidence intervals (CIs) are reported for regression models. Weighted descriptive and inferential analyses were estimated using design‐based procedures; where applicable, Cohen's d (*d*) and odds ratios (ORs) were reported as effect‐size estimates.

Algorithm‐defined MD was operationalized using a study‐specific algorithm derived from the diagnostic framework of Pope et al. (1997) and informed by the epidemiological operationalizations reported by Mitchison et al. (2022) and Ganson, Mitchison, et al. (2025). This approach was not intended to replace clinical diagnostic assessment. Rather, it was used as a transparent rule‐based self‐report classification that combines multiple components corresponding to proposed MD criteria, including muscularity‐related preoccupation, distress or impairment, and differentiation from probable ED. In line with prior algorithm‐based work, the construct was referred to as algorithm‐defined MD rather than a clinical diagnosis. Compared with a single questionnaire total‐score cutoff, this multi‐component structure is more conservative and more closely aligned with the proposed MD construct, because classification requires not only elevated muscularity‐related preoccupation but also distress or impairment and the absence of a probable ED. It nonetheless remains a self‐report approximation requiring validation against clinical assessment.

The algorithm comprised three components: criterion A (muscularity‐related preoccupation), criterion B (MD‐related distress or impairment, requiring endorsement of at least two of four indicators), and criterion C (muscularity‐focused body concern in the absence of any probable ED). Criterion A used the MDDI Drive for Size subscale, defined as a score at least 1.5 SD above the relevant distributional mean. “Relevant mean” refers to the distribution used for the respective analysis: the raw total‐sample mean for the unweighted global cutoff, raw gender‐specific means for the unweighted gender‐specific cutoffs, the weighted total‐sample mean for the weighted global cutoff, and weighted gender‐specific means for the weighted gender‐specific cutoffs. Criterion A therefore represents a relatively high‐preoccupation threshold within the corresponding distribution rather than a clinically validated cutoff. Participants meeting all three criteria were classified as having algorithm‐defined MD. Corresponding means, SDs, and cutoffs are in Table S2a; the full operationalization, including all contributing items and thresholds, is in Table S2b.

Probable ED diagnoses were derived algorithmically from self‐report data using DSM‐5‐based operationalizations informed by Mitchison et al. (2020). Based on the available questionnaire items, the study operationalized probable anorexia nervosa (AN), atypical AN, bulimia nervosa, subthreshold bulimia nervosa, binge‐eating disorder, subthreshold binge‐eating disorder, purging disorder, and unspecified/other specified eating‐disorder presentations. A binary ED variable indicating the presence of any probable ED diagnosis was used descriptively and as part of criterion C in the MD algorithm.

Because the raw online sample was younger than the adult population structure and age was relevant to algorithm‐defined MD, post‐stratification weights were calculated for the analytic sample. Weights were derived by raking to match census‐based marginal distributions for gender and age group using population counts from the 2022 German Census results database (Statistische Ämter des Bundes und der Länder 2025).

Sample characteristics were described using unweighted descriptive statistics. Algorithm‐defined MD prevalence was estimated under four conditions: unweighted/global cutoff, unweighted/gender‐specific cutoff, weighted/global cutoff, and weighted/gender‐specific cutoff. All four estimates were reported as analytic scenarios rather than treating one estimate as a definitive primary prevalence estimate. Weighted prevalence estimates and 95% CIs were computed using design‐based procedures.

To characterize algorithm‐defined MD cases descriptively, weighted group comparisons were conducted for selected sociodemographic, behavioral, and psychosocial variables. Continuous variables were compared using design‐based *t*‐tests and binary variables using design‐based chi‐square tests. Variables directly contributing to case classification were interpreted cautiously to avoid circularity.

Weighted logistic regression models were first estimated using the weighted gender‐specific algorithm‐defined MD definition as the outcome. To examine the stability of associations across analytic choices, sensitivity models repeated the pooled and men‐only regression models using alternative case‐definition and weighting specifications. Two pooled models were estimated in the male/female analytic sample. The first included gender, age, BMI, overall identity disturbance (SCIM total), attachment insecurity (VASQ insecurity), and obsessive‐compulsive symptoms (DOCS‐SF total). The second additionally included partnership status and self‐rated femininity (TMF femininity). In exploratory analyses, self‐rated masculinity was examined as an alternative specification. Because the number of female algorithm‐defined MD cases was small, no women‐only multivariable model was estimated; instead, a weighted men‐only model with the same predictor sets was estimated as a sensitivity analysis.

Sensitivity analyses examined the dependence of findings on case definition by repeating the pooled and men‐only regression models using the weighted global algorithm‐defined MD definition instead of the weighted gender‐specific definition. In addition, prevalence estimates were reported under both global and gender‐specific criterion A cutoffs in unweighted and weighted form. Additional unweighted sensitivity models were estimated using the raw gender‐specific and raw global algorithm‐defined MD definitions.

## 3 Results

### 3.1 Sample Characteristics

The full cleaned sample comprised *N* = 1468 participants, including 739 men (50.3%), 706 women (48.1%), 21 participants identifying as nonbinary or diverse (1.4%), and 2 participants who preferred not to disclose their gender (0.1%). Mean age was 31.0 years (SD = 9.82), and mean BMI was 25.1 kg/m^2^ (SD = 5.35). Men were older on average than women (31.8 [SD = 9.66] vs. 30.2 [SD = 9.99] years, *d* = 0.16, *p* = 0.003) and had a higher mean BMI (25.8 [SD = 4.91] vs. 24.2 [SD = 5.51] kg/m^2^, *d* = 0.31, *p* < 0.001). Additional sociodemographic characteristics are presented in Table 1.

**TABLE 1:** Sample characteristics of the total sample and by gender.

Variable	Total sample (*N* = 1468)	Men (*N* = 739)	Women (*N* = 706)	Nonbinary/diverse(*N* = 21)
Age, mean (SD)	31.0 (9.82)	31.8 (9.66)	30.2 (9.99)	26.5 (6.88)
Age, median [range]	28 [18–75]	30 [18–71]	27 [18–75]	24 [20–46]
BMI, mean (SD)	25.1 (5.35)	25.8 (4.91)	24.2 (5.51)	27.5 (9.20)
BMI, median [range]	24.2 [13.9–54.0]	24.7 [15.5–54.0]	22.9 [13.9–52.9]	24.2 [16.7–49.6]
Heterosexual, *n* (%)	1149 (78.3)	665 (90.0)	482 (68.3)	1 (4.8)
No migration background, *n* (%)	1066 (72.6)	533 (72.1)	516 (73.1)	16 (76.2)
Education ≥ 12 years, *n* (%)	1334 (90.9)	658 (89.0)	657 (93.1)	17 (81.0)
Single, *n* (%)	1127 (76.8)	566 (76.6)	546 (77.3)	16 (76.2)
Living in multi‐person household, *n* (%)	1019 (69.4)	510 (69.0)	495 (70.1)	13 (61.9)

*Note:* Values are based on the full cleaned sample (*N* = 1468). Two participants who preferred not to disclose their gender are included in the total sample but are not shown as a separate column. Percentages for gender‐specific columns are calculated within the respective gender group.

Abbreviation: BMI = body mass index.

For analyses involving post‐stratification weights, the analytic sample was restricted to male and female participants with complete information on the weighting variables (*N* = 1445). In the weighted analytic sample, the age distribution shifted upward relative to the raw sample (weighted mean age = 48.6 years, SE = 1.57), the gender distribution was close to balanced (48.8% men, 51.2% women), and the weighted mean BMI was 26.1 kg/m^2^ (SE = 0.43).

### 3.2 Prevalence Estimates Under Alternative Operationalizations

In the full cleaned sample, the unweighted global algorithm classified 91 of 1468 participants as algorithm‐defined MD, corresponding to a prevalence of 6.2% (95% CI 5.1–7.6). Prevalence was higher in men (81/739; 11.0%, 95% CI 8.9–13.4) than in women (10/706; 1.4%, 95% CI 0.8–2.6). Applying a gender‐specific cutoff yielded 65 algorithm‐defined MD cases overall (4.4%, 95% CI 3.5–5.6), corresponding to 6.6% (95% CI 5.1–8.7) in men and 2.3% (95% CI 1.4–3.7) in women. Any probable ED was present in 287 of 1467 participants (19.6%, 95% CI 17.6–21.7), with lower prevalence in men (11.5%, 95% CI 9.4–14.0) than in women (27.6%, 95% CI 24.4–31.0). ED subtype‐specific prevalence estimates are reported in Table S3.

In the analytic male/female sample (*N* = 1445), weighted prevalence estimates were lower than the corresponding raw‐sample estimates. With a global cutoff, weighted prevalence was 3.4% (95% CI 2.4–4.7) overall, 5.3% (95% CI 3.9–7.2) in men, and 1.5% (95% CI 0.6–4.0) in women. With gender‐specific cutoffs, weighted prevalence was 2.6% (95% CI 1.8–3.8) overall, 3.5% (95% CI 2.4–5.1) in men, and 1.7% (95% CI 0.7–4.1) in women. All four estimates are reported as analytic scenarios and are summarized in Table 2 and visualized in Figure 1.

**TABLE 2:** Prevalence of algorithm‐defined muscle dysmorphia under alternative operationalizations in the full cleaned sample and the analytic male/female sample.

Definition of cutoff	Group	Weighting	*N*	Cases	Prevalence %	95% CI
Global	Total	Unweighted	1468	91	6.20	5.08–7.55
Global	Men	Unweighted	739	81	10.96	8.91–13.42
Global	Women	Unweighted	706	10	1.42	0.77–2.59
Gender‐specific	Total	Unweighted	1468	65	4.43	3.49–5.60
Gender‐specific	Men	Unweighted	739	49	6.63	5.05–8.66
Gender‐specific	Women	Unweighted	706	16	2.27	1.40–3.65
Global	Total	Weighted	1445	92	3.36	2.42–4.65
Global	Men	Weighted	739	79	5.29	3.86–7.21
Global	Women	Weighted	706	13	1.53	0.58–4.02
Gender‐specific	Total	Weighted	1445	65	2.58	1.75–3.79
Gender‐specific	Men	Weighted	739	50	3.52	2.44–5.06
Gender‐specific	Women	Weighted	706	15	1.69	0.68–4.11

*Note:* Unweighted estimates are based on the full cleaned sample using raw‐sample cutoffs. Weighted estimates are based on the male/female analytic sample using cutoffs derived from the weighted distribution. For weighted estimates, N and cases denote unweighted sample counts. Estimates are presented as analytic scenarios; no single estimate was treated as a definitive primary prevalence estimate. As a weighting diagnostic, Kish effective sample size for the weighted analytic sample was 151.1 overall (ESS/*N* = 0.104; design effect = 9.59), with gender‐specific ESS values of 93.9 for men and 64.0 for women.

**FIGURE 1:**
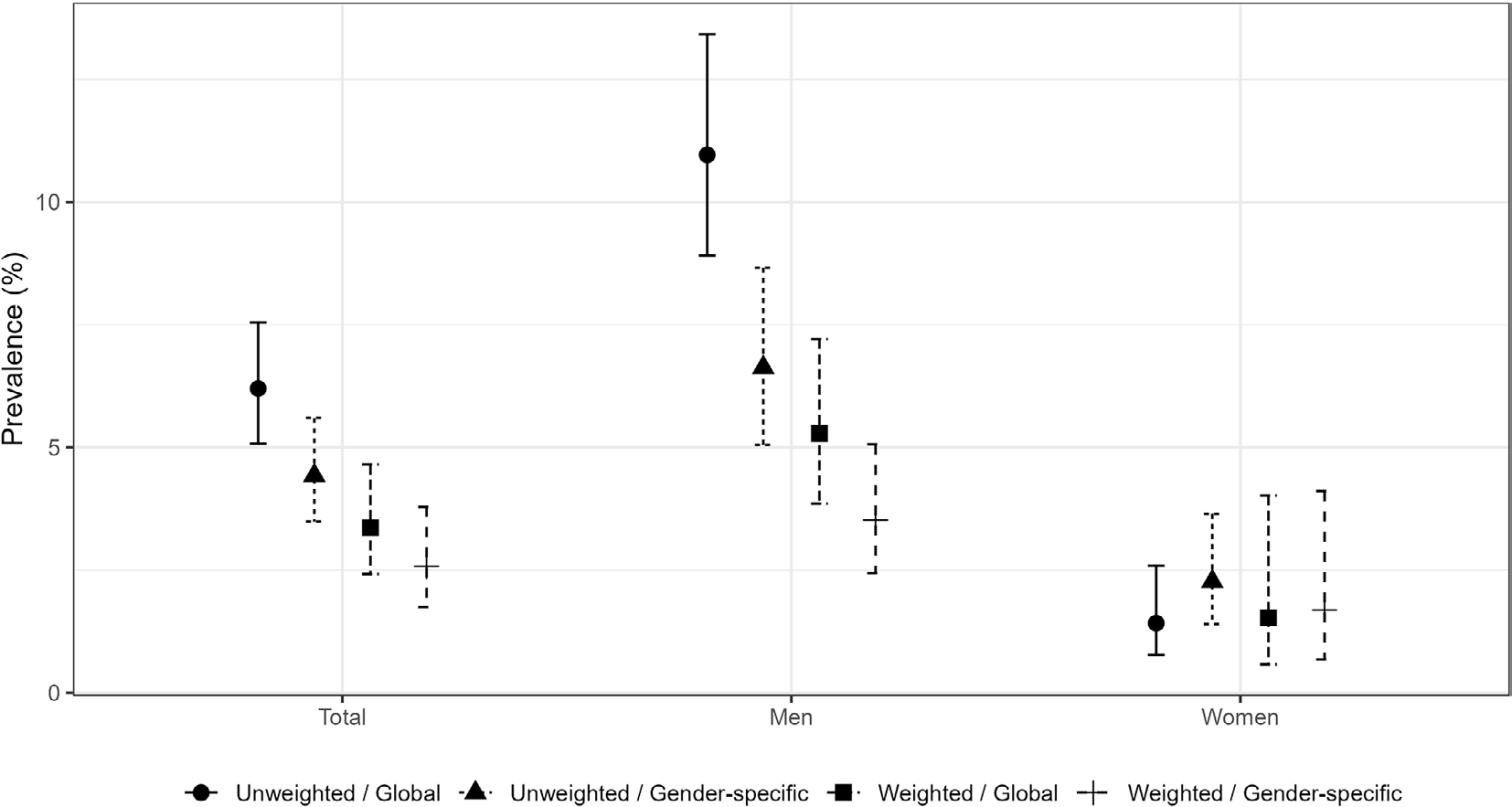
Prevalence of algorithm‐defined muscle dysmorphia under alternative cutoff and weighting specifications. *Note:* Points represent point estimates and error bars represent 95% confidence intervals. Unweighted estimates are based on the full cleaned sample using raw‐sample cutoffs. Weighted estimates are based on the male/female analytic sample using cutoffs derived from the weighted distribution.

### 3.3 Weighted Descriptive Comparisons

In weighted pooled comparisons, participants with algorithm‐defined MD were younger (35.8 vs. 48.9 years, *d* = −0.92, *p* < 0.001), had lower BMI (23.3 vs. 26.2 kg/m^2^, *d* = −0.63, *p* < 0.001), higher SCIM total scores (3.60 vs. 3.09, *d* = 0.91, *p* < 0.001), and higher DOCS‐SF scores (8.14 vs. 5.10, *d* = 0.53, *p* = 0.002). They were also more likely to report creatine use (62.4% vs. 16.4%, OR = 8.47, *p* < 0.001) and protein supplement use (88.5% vs. 54.2%, OR = 6.52, *p* < 0.001), and less likely to be partnered (13.6% vs. 40.0%, OR = 0.24, *p* = 0.001). In gender‐stratified comparisons, the same pattern was most evident among men; findings among women should be interpreted cautiously given the small number of cases. Full results are presented in Table S4.

### 3.4 Weighted Logistic Regression Models

Weighted logistic regression models using the gender‐specific algorithm‐defined MD definition showed that lower BMI (OR = 0.86, 95% CI 0.80–0.93, *p* < 0.001) and higher SCIM total scores (OR = 2.08, 95% CI 1.03–4.22, *p* = 0.042) were independently associated with algorithm‐defined MD; female gender was associated with lower odds of algorithm‐defined MD (OR = 0.33, 95% CI 0.11–1.00, *p* = 0.05). In the extended pooled model, lower BMI (OR = 0.86, 95% CI 0.80–0.93, *p* < 0.001) and higher SCIM total scores (OR = 2.19, 95% CI 1.02–4.69, *p* = 0.043) again remained independently associated with algorithm‐defined MD, and female gender remained associated with lower odds (OR = 0.27, 95% CI 0.10–0.73, *p* = 0.010). Partnership status, attachment insecurity, obsessive‐compulsive symptoms, and self‐rated femininity were not independently associated with algorithm‐defined MD. Substituting self‐rated masculinity for self‐rated femininity did not materially change the overall pattern of results, and self‐rated masculinity was likewise not independently associated with algorithm‐defined MD (OR = 1.03, 95% CI 0.78–1.35, *p* = 0.837).

In the weighted men‐only sensitivity models, younger age and lower BMI were consistently associated with algorithm‐defined MD. SCIM total was independently associated with algorithm‐defined MD in the more parsimonious model (OR = 2.10, 95% CI 1.01–4.35, *p* = 0.046) but was attenuated to trend level in the extended model (OR = 2.09, 95% CI 0.99–4.40, *p* = 0.052). Full model results are shown in Table 3, and Figure 2 displays the corresponding effect estimates and 95% confidence intervals for the two weighted pooled models.

**TABLE 3:** Weighted logistic regression models predicting algorithm‐defined muscle dysmorphia using the gender‐specific outcome definition.

Predictor	Model 1 OR [95% CI]	*p*	Model 2 OR [95% CI]	*p*
Female gender	0.33 [0.11, 1.00]	0.050	0.27 [0.10, 0.73]	0.010
Age	0.97 [0.94, 1.00]	0.086	0.99 [0.94, 1.03]	0.542
BMI	0.86 [0.80, 0.93]	< 0.001	0.86 [0.80, 0.93]	< 0.001
SCIM total	2.08 [1.03, 4.22]	0.042	2.19 [1.02, 4.69]	0.043
VASQ insecurity	1.02 [0.95, 1.09]	0.651	1.01 [0.94, 1.09]	0.749
DOCS‐SF total	1.02 [0.97, 1.08]	0.434	1.02 [0.97, 1.08]	0.387
Partnered	—	—	0.37 [0.08, 1.62]	0.185
TMF femininity	—	—	1.08 [0.83, 1.39]	0.579

*Note:* Results are based on weighted logistic regression models in the male/female analytic sample using the weighted gender‐specific algorithm‐defined MD definition as the outcome.

Abbreviations: BMI = body mass index; CI = confidence interval; DOCS‐SF = Dimensional Obsessive‐Compulsive Scale–Short Form; OR = odds ratio; SCIM = Self‐Concept and Identity Measure; TMF = Traditional Masculinity‐Femininity scale; VASQ = Vulnerable Attachment Style Questionnaire.

**FIGURE 2:**
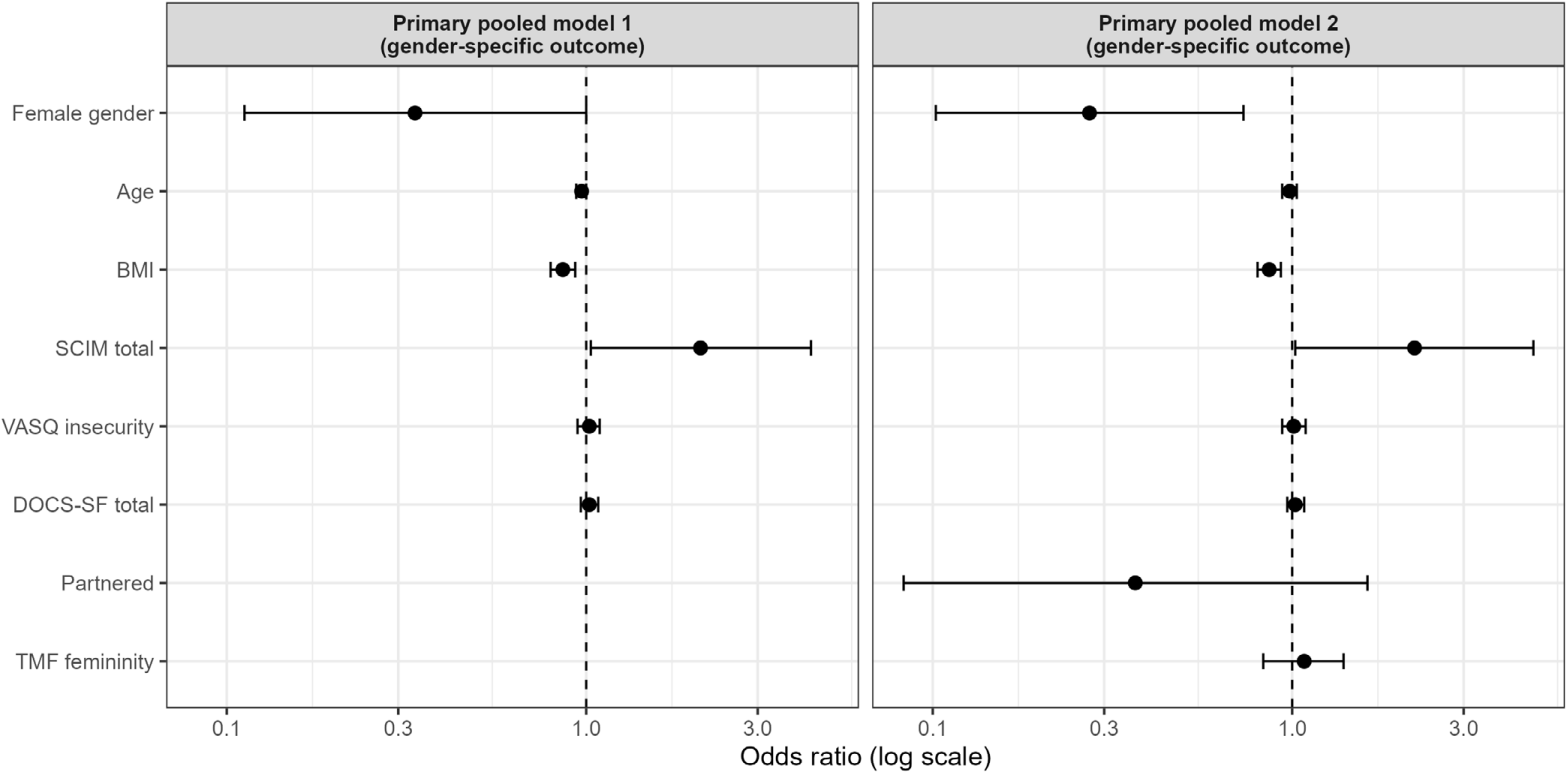
Weighted pooled logistic regression models predicting algorithm‐defined muscle dysmorphia using the gender‐specific outcome definition. *Note:* Points represent odds ratios and horizontal bars represent 95% confidence intervals. Model 1 included gender, age, BMI, SCIM total, VASQ insecurity, and DOCS‐SF total. Model 2 additionally included partnership status and TMF femininity.

### 3.5 Sensitivity Analyses

When the regression models were repeated using alternative case definitions and unweighted raw‐sample specifications—based on the raw gender‐specific and raw global case definitions, corresponding to the two unweighted prevalence scenarios—the overall association pattern was broadly similar. Lower BMI and higher SCIM total scores were the most consistent correlates of algorithm‐defined MD, and female gender showed lower odds in pooled models, particularly under the global cutoff. Age, attachment insecurity, obsessive‐compulsive symptoms, partnership status, and self‐rated gender‐role characteristics were not consistently associated. Thus, although algorithm‐defined prevalence estimates varied across operationalizations, the main correlational pattern was comparatively stable. Full results are in Table S5.

## 4 Discussion

### 4.1 Principal Findings

The present study showed that self‐report algorithm‐defined estimates of MD in a gender‐balanced adult online sample varied substantially across weighting and case‐definition strategies. No single estimate should be interpreted as a definitive prevalence estimate. Despite variation, lower BMI and higher SCIM total scores emerged as the most consistent correlates across regression specifications.

### 4.2 Prevalence Estimates, Weighting, and Case Definition

The prevalence estimates observed in the present study should be interpreted against the marked methodological heterogeneity of the MD literature. Compared with high risk or fitness‐oriented samples, the prevalence rates reported here were clearly lower than those reported by Zeeck et al. (2018), who identified elevated proportions of participants “at risk” for MD in a German fitness‐oriented community sample. This difference is plausible because Zeeck et al. relied on questionnaire‐based cutoffs rather than an algorithm‐based approximation of probable diagnosis and did not exclude overlapping ED symptomatology. Similarly, earlier work in bodybuilders, weightlifters, and related high‐risk groups has often reported substantially higher prevalence estimates than those observed in broader community samples (Lechner et al. 2019; Nieuwoudt et al. 2015).

At the same time, the raw global prevalence estimate among men in the present study (11.0%) was higher than the lower and more convergent estimates reported in more recent population‐based studies from Australia and North America (Ganson, Mitchison, et al. 2025; Mitchison et al. 2022). In our view, this discrepancy likely reflects both sampling and operationalization. First, the raw online sample was relatively young, and younger age was associated with algorithm‐defined MD in the present data. Once age and gender were incorporated into the weighting procedure, prevalence estimates were reduced substantially, suggesting that unweighted online samples may overstate population‐oriented prevalence when younger adults are overrepresented.

Second, prevalence estimates were sensitive to how criterion A was defined. Because the threshold was based on the sample distribution of the MDDI Drive for Size subscale, it was inherently dependent on which distribution was used. In the present data, moving from a global to a gender‐specific cutoff reduced the male prevalence estimate and slightly increased the female estimate. This pattern raises the possibility that a single global cutoff in mixed‐gender samples may overclassify algorithm‐defined MD among men while underclassifying it among women. The present data do not establish that gender‐specific cutoffs are definitively correct or universally preferable. However, they do suggest that transferring a threshold primarily used in male or male‐dominated samples to a mixed adult sample should not be treated as self‐evident. Accordingly, the weighted gender‐specific estimate should be interpreted as one population‐adjusted scenario rather than as a definitive primary prevalence estimate. More broadly, these findings suggest that mixed‐gender analyses of MD require greater methodological care.

A further factor specific to the female estimate concerns the criterion C eating‐disorder exclusion. Because algorithm‐defined MD classification required the absence of any probable ED, participants with a probable ED were removed before MD classification. In the present sample, probable EDs were considerably more frequent among women than men (27.6% vs. 11.5%), so this exclusion removed a proportionally larger share of women. The comparatively low female estimate therefore reflects not only the relative criterion A threshold but also this clinically motivated exclusion. This is a deliberate and, in our view, appropriate feature of the algorithm rather than a bias, as it reduces overlap with thinness‐oriented eating pathology; we note it here because it bears directly on the interpretation of the observed gender difference in algorithm‐defined MD.

### 4.3 Sociodemographic, Behavioral, and Psychological Correlates

Regarding correlates, the most robust finding across regression specifications was the inverse association between BMI and algorithm‐defined MD. This pattern may reflect the body‐related concerns characteristic of MD, but it should be interpreted cautiously because BMI is an imprecise marker that does not distinguish between lean mass and fat mass (Rothman 2008). Rather than indicating a simple body‐size effect, the finding may point to the relevance of a perceived mismatch between one's current body and a desired muscular, lean physique.

A second notable finding was the independent association between algorithm‐defined MD and overall identity disturbance, assessed with the SCIM, which captures clinically relevant disturbances in self‐concept and identity (Kaufman et al. 2015). In the weighted pooled models, SCIM total was significantly associated with algorithm‐defined MD even after adjustment for gender, age, BMI, attachment insecurity, and obsessive‐compulsive symptoms. In the men‐only models, this association remained evident, although it was somewhat attenuated in the extended model. Under the global‐cutoff sensitivity analyses, the association became even clearer. This pattern is also consistent with prior work linking MD symptoms to self‐concept, self‐esteem, and broader psychosocial vulnerabilities (Bégin et al. 2019; González‐Martí et al. 2014; Olivardia et al. 2004; Rodrigue et al. 2018). Nevertheless, the present data do not justify treating identity disturbance as a causal mechanism. Because the study is cross‐sectional, difficulties related to identity may precede MD‐related symptoms, follow them, or reflect overlapping vulnerability. The consistency of the association across models suggests that identity‐related impairment deserves greater attention in future MD research.

By contrast, attachment insecurity and obsessive‐compulsive symptoms did not emerge as independent correlates in the multivariable models. Additional exploratory analyses further suggested that neither self‐rated femininity nor self‐rated masculinity was independently associated with algorithm‐defined MD. This null finding contrasts with prior studies linking conformity to masculine gender norms and masculine discrepancy stress to MD symptoms or MD risk (Ganson et al. 2024; Grunewald et al. 2022). One possible explanation is that the present study assessed self‐rated masculinity and femininity rather than conformity to specific masculine norms, such as self‐reliance, emotional control, or dominance. Behaviorally, algorithm‐defined MD cases were substantially more likely to report creatine use and protein supplement use, suggesting greater muscularity‐oriented behavioral investment in this group. Female gender was associated with lower odds of algorithm‐defined MD in the pooled models, whereas younger age was more consistently associated with algorithm‐defined MD in men‐only and global‐cutoff analyses. These findings should be interpreted cautiously but suggest that algorithm‐defined MD may be particularly relevant among younger men under some operationalizations.

### 4.4 Implications for Conceptual Models and Future Epidemiology

The present findings have implications for both conceptual models of MD and future epidemiological practice. Conceptually, the observed pattern suggests that algorithm‐defined MD in the general population may involve body‐related discrepancy, muscularity‐oriented behavioral investment, and vulnerability related to identity. This interpretation is broadly consistent with recent network‐analytic research in men suggesting that muscularity concerns are embedded in a broader symptom structure of disordered eating and body‐shaping behaviors rather than representing an isolated phenomenon (Eschrich et al. 2025). At a broader level, these findings align with transdiagnostic and sociocultural approaches to body image psychopathology, while also suggesting that not all theoretically relevant psychosocial variables remain equally informative once overlapping variance is considered (Bégin et al. 2019; Rica and Sepúlveda 2024).

The tripartite influence model may provide a useful framework for future research on sociocultural pathways to algorithm‐defined MD, particularly regarding peer and media influences and the internalization of appearance ideals (Tylka 2011). Methodologically, the study highlights the need for greater transparency in operationalizing algorithm‐defined MD. Because self‐report algorithmic approaches are increasingly used to generate MD prevalence estimates yet yield results that vary substantially with operational and sampling decisions, their methodological implications require direct empirical evaluation. Future studies should specify whether criterion A was derived from a global or gender‐specific distribution, whether estimates were weighted, and how ED exclusions were handled, as these decisions may meaningfully shape conclusions. Beyond transparent reporting, the field would benefit from a consensus approach for assessing MD in epidemiological samples, including how muscularity‐related preoccupation, clinical significance, and ED overlap should be operationalized and validated.

### 4.5 Limitations and Strengths

Several limitations should be considered. First, the cross‐sectional design precludes causal or temporal inference. Second, all measures were self‐reported. Third, although Prolific allowed recruitment of a large community‐based adult sample, online panels may introduce self‐selection bias, and post‐stratification weighting adjusts only for measured demographic differences, not unmeasured selection. Fourth, although weighting improved demographic alignment with the German adult population, the unequal weights reduced the effective precision of weighted estimates; the weighted gender‐specific estimate should therefore be read as a population‐adjusted scenario rather than a definitive estimate. Fifth, algorithm‐defined MD was identified by self‐report rather than clinical interview and should be understood as an epidemiological approximation rather than a confirmed diagnosis; the findings do not establish the algorithm's diagnostic validity but highlight the need for clinically validated, consensus‐based methods. Relatedly, criterion A relied on distributional thresholds from the MDDI Drive for Size subscale, which ensures comparability with recent algorithm‐based work but provides no externally or clinically validated cutoff; it should therefore be read as identifying relatively high muscularity‐related preoccupation within the analytic distribution rather than establishing caseness by itself. Sixth, weighted analyses were restricted to male and female participants, as other gender categories were too small for stable estimation; transgender status and sex assigned at birth were not assessed, precluding analysis of transgender and gender‐diverse participants, who may show distinct MD presentations and prevalence and should be included or oversampled in future work. Seventh, the number of female algorithm‐defined MD cases was modest, limiting female‐specific inference. Finally, criterion C conservatively excluded participants with any probable ED diagnosis, reducing phenomenological overlap but possibly limiting comparability with studies using narrower exclusion rules.

Nonetheless, the study has several important strengths. First, to our knowledge it is among the first studies to demonstrate directly how strongly algorithm‐defined MD estimates in a mixed adult sample depend on case‐definition and weighting decisions, with overall estimates ranging from 2.6% to 6.2% and the male‐to‐female ratio varying from approximately 2:1 to 8:1 in the same data; this sensitivity is itself a substantive finding for a literature that has typically reported single estimates. Second, the sample was large and gender‐balanced (50.3% men, 48.1% women), which is uncommon in MD epidemiology, where most prevalence work has been conducted in boys, men, or male‐dominated samples. This balance allowed us to derive female estimates of algorithm‐defined MD, including weighted estimates, which remain rare in a field that has historically centered on men. Third, the study assessed probable EDs alongside algorithm‐defined MD and applied a conservative exclusion rule to reduce overlap with thinness‐oriented eating pathology. Finally, sensitivity analyses strengthened interpretability by showing which conclusions were robust across alternative case definitions, even as the prevalence estimates themselves varied.

### 4.6 Clinical and Public Health Implications and Conclusion

Self‐report algorithm‐defined MD may affect a meaningful minority of adults and should not be viewed solely as a niche problem of narrowly defined fitness subcultures. The findings further suggest that epidemiological estimates depend on operationalization in mixed samples and that identity‐related difficulties may warrant greater attention in assessment and theory.

In conclusion, the present study suggests that prevalence estimates of algorithm‐defined MD in mixed adult samples are strongly influenced by both weighting strategy and criterion A operationalization. In particular, the findings raise the possibility that a single global cutoff may not be optimal when men and women are analyzed together. Future epidemiological studies should therefore report these decisions transparently, specify clearly how criterion A was defined, and evaluate whether gender‐specific thresholds improve interpretability in mixed‐gender samples.

## Author Contributions

**Christopher Zaiser:** conceptualization, investigation, methodology, writing – original draft, writing – review and editing, visualization, formal analysis. **Georg Halbeisen:** methodology, data curation, formal analysis, validation, writing – review and editing, conceptualization. **Georgios Paslakis:** supervision, resources, conceptualization, investigation, writing – review and editing. **Marietta Lieb:** writing – review and editing, methodology. **Nora M. Laskowski:** conceptualization, writing – review and editing, project administration, investigation, data curation, methodology.

## Lived Experience Involvement Statement

No specific efforts were undertaken to involve persons with lived experience in the study design or execution, or in the preparation of this manuscript.

## Funding

The authors have nothing to report.

## Ethics Statement

The study was approved by the Ethics Committee of the Medical Faculty of Ruhr‐University Bochum (approval date: 16 April 2025; reference: “EK‐Antrag MD Prolific v1.0”). All participants provided electronic informed consent before participation. All procedures were conducted in accordance with the Declaration of Helsinki.

## Conflicts of Interest

The authors declare no conflicts of interest.

## Supporting information


**Table S1:** Descriptive statistics and internal consistencies of study measures in the total sample and by gender.
**Table S2a:** Raw‐sample and weighted analytic‐sample cutoffs for MDDI drive for size.
**Table S2b:** Operationalization of algorithm‐defined muscle dysmorphia (MD) and probable eating disorder (ED) diagnoses.
**Table S3:** Prevalence of probable eating disorder subtypes in the total unweighted sample and by gender.
**Table S4:** Weighted descriptive comparisons by algorithm‐defined muscle dysmorphia (MD) status using the weighted gender‐specific algorithm‐defined MD outcome.
**Table S5:** Unweighted sensitivity regression models using raw gender‐specific and raw global case definitions.

## Data Availability

The data that support the findings of this study are not publicly available due to ethical and data protection considerations related to participant confidentiality. De‐identified data and analysis code may be made available from the corresponding author upon reasonable request, subject to applicable ethical and legal restrictions.
