# Supplementary material for: Algorithm‐Defined Muscle Dysmorphia Estimates Across Weighting and Case‐Definition Strategies in a Gender‐Balanced Adult Online Sample
Source: Int J Eat Disord. 2026 Jul 9;59(11):2462–72. doi: 10.1002/eat.70170 (PMC13726458; doi:10.1002/eat.70170)
Supplement: Supplementary file 1 [file EAT-59-2462-s001.docx]

| **Instrument / scale** | **Total, *M* (*SD*)** | ***α*** | **Men, *M* (*SD*)** | ***α*** | **Women, *M* (*SD*)** | ***α*** |
| --- | --- | --- | --- | --- | --- | --- |
| DMS total | 2.53 (0.92) | .91 | 2.84 (0.96) | .91 | 2.21 (0.75) | .87 |
| DOCS-SF total | 6.51 (5.69) | .90 | 6.46 (5.51) | .89 | 6.53 (5.81) | .91 |
| EDE-Q shape concern | 2.29 (1.65) | .92 | 1.91 (1.49) | .91 | 2.68 (1.71) | .92 |
| EDE-Q weight concern | 1.88 (1.57) | .85 | 1.51 (1.35) | .82 | 2.25 (1.68) | .86 |
| EDS total | 10.50 (7.19) | .88 | 10.20 (7.47) | .89 | 10.60 (6.67) | .86 |
| K10 total | 23.40 (8.06) | .92 | 21.80 (7.72) | .92 | 24.80 (8.03) | .92 |
| MDDI drive for size | 11.10 (4.59) | .86 | 12.80 (4.71) | .87 | 9.33 (3.74) | .79 |
| MDDI total | 28.40 (8.26) | .82 | 29.60 (8.73) | .84 | 27.00 (7.51) | .80 |
| SCIM consolidated identity | 3.46 (0.58) | .35 | 3.43 (0.59) | .38 | 3.49 (0.56) | .33 |
| SCIM disturbed identity | 3.11 (1.16) | .83 | 3.08 (1.13) | .82 | 3.15 (1.19) | .85 |
| SCIM lack of identity | 3.33 (0.87) | .55 | 3.38 (0.87) | .55 | 3.29 (0.87) | .55 |
| SCIM total | 3.36 (0.61) | .78 | 3.35 (0.58) | .75 | 3.37 (0.64) | .80 |
| TMF femininity | 3.73 (1.67) | .95 | 2.42 (1.09) | .91 | 5.08 (0.94) | .85 |
| TMF masculinity | 3.63 (1.53) | .94 | 4.81 (1.00) | .87 | 2.41 (0.93) | .84 |
| VASQ insecurity | 31.90 (7.48) | .82 | 31.80 (7.32) | .81 | 32.00 (7.68) | .83 |
| VASQ proximity-seeking | 29.20 (5.59) | .73 | 28.30 (5.29) | .69 | 30.20 (5.70) | .75 |
| VASQ total | 61.20 (9.16) | .74 | 60.00 (8.73) | .71 | 62.30 (9.52) | .76 |

**Supplementary Table S1**

*Descriptive statistics and internal consistencies of study measures in the total sample and by gender.*

**Note.** Total sample $N=1,468$, men $N=739$, women $N=706$. Ns may vary slightly across measures because of missing values. Dashes indicate that no internal consistency estimate was reported for these scale scores in the present table.

**Supplementary Table S2a**

*Raw-sample and weighted analytic-sample cutoffs for MDDI drive for size.*

| **Framework** | **Group** | **Mean** | ***SD*** | **Cutoff (Mean + 1.5 *SD*)** |
| --- | --- | --- | --- | --- |
| Unweighted raw sample | Total | 11.10 | 4.59 | 18.00 |
| Unweighted raw sample | Men | 12.80 | 4.71 | 19.80 |
| Unweighted raw sample | Women | 9.33 | 3.74 | 14.90 |
| Weighted analytic sample | Total | 10.40 | 4.55 | 17.20 |
| Weighted analytic sample | Men | 11.50 | 4.99 | 19.00 |
| Weighted analytic sample | Women | 9.36 | 3.80 | 15.10 |

**Note.** Weighted cutoffs were derived from the weighted analytic sample restricted to male and female participants.

**Supplementary Table S2b**

*Operationalization of algorithm-defined muscle dysmorphia (MD) and probable eating disorder (ED) diagnoses.*

| **Component** | **Operationalization** |
| --- | --- |
| **A. Algorithm-defined muscle dysmorphia** | |
| Criterion A (global, weighted) | MDDI Drive for Size score ≥ [weighted] global cutoff (defined as 1.5 SD above the [weighted] mean) |
| Criterion A (gender-specific, weighted) | MDDI Drive for Size score ≥ [weighted] gender-specific cutoff (defined as 1.5 SD above the [weighted] gender-specific mean) |
| Criterion B1: functional interference | (dms_12 ≥ 4) or (mddi_11 ≥ 4) or (mddi_13 ≥ 4) |
| Criterion B2: body exposure distress | (edeq_28 ≥ 4) |
| Criterion B3: psychological distress | (K10_total > 15) |
| Criterion B4: persistence of muscularity-oriented behavior | (dms_2 ≥ 4) or (edeq_p1 ≥ 12) or (edeq_p2 ≥ 1) |
| Criterion B overall | At least 2 of the 4 B indicators fulfilled |
| Criterion C | (dms_1 ≥ 4) and no probable eating-disorder diagnosis |
| Weighted gender-specific outcome | Algorithm-defined MD = weighted gender-specific criterion A + criterion B + criterion C |
| Weighted global outcome | Algorithm-defined MD = weighted global criterion A + criterion B + criterion C |
| **B. Probable eating-disorder algorithm** | |
| Diagnosis | Operationalization |
| AN | (BMI ≤ 18.5) and ((extreme_ctrl_persist or fear_fat)) and overevaluation |
| BN | (OBE_4plus) and extreme control persistence and overevaluation, excluding AN |
| BED | (OBE_4plus), (≥ 3) binge features, distress item endorsed, no extreme control, excluding AN/BN |
| AAN | (BMI > 18.5), weight loss (> 0), ((extreme_ctrl_persist or fear_fat)), and overevaluation, excluding AN/BN/BED |
| SBN | (OBE_2plus), extreme control at lower threshold, and overevaluation, excluding higher-order ED categories |
| SBED | (OBE_2plus), (≥ 3) binge features, distress item endorsed, no extreme control, excluding higher-order ED categories |
| PD | Purging present without extreme control persistence, excluding higher-order ED categories |
| UFED | ((OBE_2plus or extreme_ctrl_persist)), overevaluation, and (K10 ≥ 15), excluding higher order ED categories |
| Any probable ED | Presence of any ED subtype above |

**Note.** MD = muscle dysmorphia; ED = eating disorder; MDDI = Muscle Dysmorphic Disorder Inventory; DMS = Drive for Muscularity Scale; K10 = Kessler Psychological Distress Scale; BMI = body mass index; AN = anorexia nervosa; BN = bulimia nervosa; BED = binge-eating disorder; AAN = atypical anorexia nervosa; SBN = subthreshold bulimia nervosa; SBED = subthreshold binge-eating disorder; PD = purging disorder; UFED = unspecified feeding or eating disorder. The derived variables extreme_ctrl_persist, fear_fat, and overevaluation indicate persistent extreme weight-control behaviours, fear of weight gain/becoming fat, and overevaluation of weight/shape, respectively. OBE_4plus and OBE_2plus denote objective binge-eating frequency thresholds used in the diagnostic algorithm (≥ 4 and ≥ 2 episodes, respectively), and binge features denotes the associated binge-eating features required for binge-eating disorder classifications. The full coding logic followed the algorithmic implementation used in the analytic workflow.

**Supplementary Table S3**

*Prevalence of probable eating disorder subtypes in the total unweighted sample and by gender.*

| **ED subtype** | **Total, *N* (%) [95% CI]** | **Men, *N* (%) [95% CI]** | **Women, *N* (%) [95% CI]** |
| --- | --- | --- | --- |
| AN | 8 (0.54) [0.28, 1.07] | 1 (0.14) [0.02, 0.76] | 7 (0.99) [0.48, 2.03] |
| AAN | 63 (4.29) [3.37, 5.45] | 15 (2.03) [1.23, 3.32] | 46 (6.52) [4.92, 8.58] |
| BN | 85 (5.79) [4.71, 7.10] | 32 (4.33) [3.08, 6.05] | 51 (7.22) [5.54, 9.37] |
| BED | 52 (3.54) [2.71, 4.62] | 17 (2.30) [1.44, 3.66] | 33 (4.67) [3.35, 6.49] |
| Other/remaining ED categories | 82 (5.59) [4.53, 6.89] | 20 (2.71) [1.76, 4.15] | 61 (8.64) [6.79, 10.90] |
| Any probable ED | 287 (19.60) [17.60, 21.70] | 85 (11.50) [9.41, 14.00] | 195 (27.60) [24.40, 31.00] |

**Note.** ED = eating disorder; N = number of cases; CI = confidence interval; AN = anorexia nervosa; AAN = atypical anorexia nervosa; BN = bulimia nervosa; BED = binge-eating disorder; SBN = subthreshold bulimia nervosa; SBED = subthreshold binge-eating disorder; PD = purging disorder; UFED = unspecified feeding or eating disorder. “Other/remaining ED categories” combines subthreshold bulimia nervosa, subthreshold binge-eating disorder, purging disorder, and unspecified feeding or eating disorder. Percentages are reported within the total sample and within each gender group, respectively.

**Supplementary Table S4**

*Weighted descriptive comparisons by algorithm-defined* *muscle dysmorphia (MD) status using the weighted gender-specific algorithm-defined* *MD outcome.*

| **Variable** | **No algorithm-defined** **MD** | **Algorithm-defined** **MD** | **Test statistic** | ***p*** |
| --- | --- | --- | --- | --- |
| **A. Total weighted analytic sample** | | | | |
| Age | 48.91 (1.58) | 35.79 (3.30) | -3.59 | < .001 |
| BMI | 26.16 (0.44) | 23.31 (0.41) | -4.77 | < .001 |
| DOCS-SF total | 5.10 (0.40) | 8.14 (0.87) | 3.17 | .002 |
| SCIM total | 3.09 (0.04) | 3.60 (0.07) | 5.93 | < .001 |
| TMF femininity | 3.61 (0.12) | 3.44 (0.32) | -0.49 | .621 |
| VASQ insecurity | 30.90 (0.46) | 33.78 (1.47) | 1.86 | .062 |
| Creatine use, % | 16.36 (2.32) | 62.37 (8.00) | 43.74 | < .001 |
| Partnered, % | 39.96 (4.28) | 13.61 (5.17) | 10.84 | .001 |
| Protein supplement use, % | 54.24 (4.22) | 88.53 (4.51) | 20.38 | < .001 |
| **B. Men only** | | | | |
| Age | 47.03 (1.97) | 32.38 (1.04) | -6.57 | < .001 |
| BMI | 27.36 (0.40) | 24.05 (0.39) | -5.92 | < .001 |
| DOCS-SF total | 5.07 (0.45) | 7.85 (1.03) | 2.47 | .014 |
| SCIM total | 3.14 (0.06) | 3.63 (0.10) | 4.22 | < .001 |
| TMF femininity | 2.27 (0.09) | 2.67 (0.15) | 2.25 | .025 |
| VASQ insecurity | 30.49 (0.47) | 34.54 (1.11) | 3.36 | < .001 |
| Creatine use, % | 25.46 (4.22) | 62.76 (7.70) | 19.35 | < .001 |
| Partnered, % | 47.23 (5.49) | 15.92 (6.41) | 10.44 | .001 |
| Protein supplement use, % | 52.36 (5.40) | 86.17 (5.50) | 14.58 | < .001 |
| **C. Women only** | | | | |
| Age | 50.65 (2.35) | 42.56 (7.08) | -1.08 | .279 |
| BMI | 25.03 (0.71) | 21.82 (1.22) | -2.27 | .024 |
| DOCS-SF total | 5.12 (0.65) | 8.74 (1.73) | 1.96 | .051 |
| SCIM total | 3.04 (0.07) | 3.54 (0.08) | 4.94 | < .001 |
| TMF femininity | 4.86 (0.12) | 4.96 (0.28) | 0.34 | .737 |
| VASQ insecurity | 31.29 (0.77) | 32.28 (3.29) | 0.29 | .770 |
| Creatine use, % | 7.88 (1.40) | 61.60 (18.61) | 24.09 | < .001 |
| Partnered, % | 33.18 (6.17) | 9.03 (7.63) | 3.39 | .066 |
| Protein supplement use, % | 55.99 (6.39) | 93.24 (6.99) | 6.83 | .009 |

**Note.** Values for continuous variables are weighted mean (SE); values for binary variables are weighted percentage (SE). The outcome was algorithm-defined MD based on the weighted gender-specific criterion A definition.

**Supplementary Table S5**

*Unweighted sensitivity regression models using raw gender-specific and raw global case definitions.*

| **Predictor** | **Raw gender-specific M1 OR [95% CI], p** | **Raw gender-specific M2 OR [95% CI], p** | **Raw global M1 OR [95% CI], p** | **Raw global M2 OR [95% CI], p** |
| --- | --- | --- | --- | --- |
| Female gender | 0.21 [0.11, 0.38], p < .001 | 0.25 [0.11, 0.57], p = .001 | 0.08 [0.04, 0.16], p < .001 | 0.08 [0.03, 0.18], p < .001 |
| Age | 1.03 [1.00, 1.06], p = .017 | 1.04 [1.01, 1.07], p = .006 | 1.00 [0.97, 1.02], p = .887 | 1.01 [0.98, 1.03], p = .703 |
| BMI | 0.82 [0.75, 0.89], p < .001 | 0.82 [0.76, 0.89], p < .001 | 0.89 [0.83, 0.94], p < .001 | 0.89 [0.84, 0.95], p < .001 |
| SCIM total | 1.83 [1.11, 3.01], p = .018 | 1.87 [1.13, 3.08], p = .014 | 1.95 [1.26, 3.02], p = .003 | 1.94 [1.25, 3.01], p = .003 |
| VASQ insecurity | 1.03 [0.99, 1.07], p = .221 | 1.02 [0.98, 1.07], p = .274 | 1.01 [0.98, 1.05], p = .474 | 1.01 [0.98, 1.05], p = .523 |
| DOCS-SF total | 1.04 [0.99, 1.09], p = .137 | 1.04 [0.99, 1.09], p = .128 | 1.04 [0.99, 1.08], p = .114 | 1.04 [0.99, 1.08], p = .110 |
| Partnered | — | 0.56 [0.23, 1.25], p = .181 | — | 0.61 [0.27, 1.24], p = .194 |
| TMF femininity | — | 0.93 [0.73, 1.18], p = .552 | — | 1.04 [0.84, 1.27], p = .736 |

**Note.** M1 included gender, age, BMI, SCIM total, VASQ insecurity, and DOCS-SF total. M2 additionally included partnership status and self-rated femininity. Raw gender-specific models used the unweighted gender-specific algorithm-defined MD definition; raw global models used the unweighted global algorithm-defined MD definition. Pooled models included n = 1,444 participants; events were 65 for the raw gender-specific definition and 91 for the raw global definition.
